# Conventional alpha beta (αβ) T cells do not contribute to acute intestinal ischemia-reperfusion injury in mice

**DOI:** 10.1371/journal.pone.0181326

**Published:** 2017-07-13

**Authors:** Yi Yu, Xiaoyan Feng, Gertrud Vieten, Stephanie Dippel, Tawan Imvised, Faikah Gueler, Benno M. Ure, Jochen F. Kuebler, Christian Klemann

**Affiliations:** 1 Department of Pediatric Surgery, Center of Surgery, Hannover Medical School, Hanover, Germany; 2 Department of Nephrology, Hannover Medical School, Hanover, Germany; 3 Department of Pediatric Pneumology, Allergology and Neonatology, Hannover Medical School, Hanover, Germany; National Institutes of Health, UNITED STATES

## Abstract

**Purpose:**

Ischemia-reperfusion injury (IRI) is associated with significant patient mortality and morbidity. The complex cascade of IRI is incompletely understood, but inflammation is known to be a key mediator. In addition to the predominant innate immune responses, previous research has also indicated that αβ T cells contribute to IRI in various organ models. The aim of this study was to clarify the role αβ T cells play in IRI to the gut.

**Methods:**

Adult *wild-type* (WT) and *αβ T cell-deficient* mice were subjected to acute intestinal IRI with 30min ischemia followed by 4h reperfusion. The gene expression of pro-inflammatory cytokines was measured by qPCR, and the influx of leukocyte subpopulations in the gut was assessed via flow cytometry and histology. Pro-inflammatory cytokines in the serum were measured, and transaminases were assessed as an indicator of distant organ IRI.

**Results:**

Intestinal IRI led to an increased expression of pro-inflammatory cytokines in the gut tissue and an influx of leukocytes that predominantly consisted of neutrophils and macrophages. Furthermore, intestinal IRI increased serum IL-6, TNF-α, and ALT/AST levels. The αβ T cell-deficient mice did not exhibit a more significant increase in pro-inflammatory cytokines in the gut or serum following IR than the WT mice. There was also no difference between WT- and αβ T cell-deficient mice in terms of neutrophil infiltration or macrophage activation. Furthermore, the increase in transaminases was equal in both groups indicating that the level of distant organ injury was comparable.

**Conclusion:**

An increasing body of evidence demonstrates that αβ T cells play a key role in IRI. In the gut, however, αβ T cells are not pivotal in the first hours following acute IRI as deficiency does not impact cytokine production, neutrophil recruitment, macrophage activation, or distant organ injury. Thus, αβ T cells may be considered innocent bystanders during the acute phase of intestinal IRI.

## Introduction

Ischemia reperfusion injury (IRI) is a significant clinical problem that impacts many organ systems, including the kidneys, brain, heart, liver, lungs, and intestine. In addition to being inevitable in organ transplantation, IRI has implications for the pathogenesis of infarction, stroke, and shock-mediated pathology following the acute injury of the liver, kidneys and ischemia gut, the latter of which includes necrotizing enterocolitis (NEC) [1–4].

The complex interplay of endothelium, immune cells, and cytokine release following local cellular hypoxia results in an inflammatory response and subsequent IRI [1]. The cells and components of the innate immune system, including neutrophils, macrophages, and secretion of inflammatory cytokines/chemokines, and the activation of the complement system, have been firmly established as pivotal in IRI [1,5–8]. Recent experimental studies that have examined the pathogenesis of IRI have found that mediators of inflammation other than the innate immune cells play a fundamental role in the development of IRI and have demonstrated the important contributions of conventional T cells in the development of this condition [9–12]. T and B cells constitute the main arms of the adaptive immune response. Initially regarded as “passive observers”, T cells are now broadly recognized as effector and/or regulatory cells in the pathogenesis of IRI. T cells have been found to act as mediators in the pathogenesis of for IRI in the brain, lung, heart, liver, and kidney in mice models [9–12]. In contrast, the role of T cells in intestinal IRI is questionable. The few published studies that have assessed lymphocytes in intestinal IRI have demonstrated that various T cell subpopulations infiltrate the gut following IR [13]. Functional studies on SCID mice or mice treated with anti-thymocyte globulin (ATG) suggested an amelioration of intestinal IRI by lack of T cells or their inhibition, respectively [14,15]. However, SCID mice also lack non-conventional γδ T cells, B cells, and the immunoglobulins that are crucial for complement activation, while ATG has a broad range of targets apart from T cells [16]. Thus, the purpose of the current study was to clarify the role αβ T cells play in the development of intestinal IRI. Through utilizing a mutant mouse strain that selectively lacks αβ T cells, we demonstrated that this lymphocyte population does not have a critical impact on the inflammation in the first few hours following acute intestinal ischemia-reperfusion.

## Materials and methods

### Animals and model of intestinal IRI

Four-week-old male *C57BL/6J wild-type* (WT) mice and αβ T cell deficient (*B6*._*129*_*S*_*2*_*-Tcra*^*tm1Mo*^m/J) mice (mean body-weight (BW) 15g) were used in all experiments [17]. All procedures were approved by the local animal welfare committee *Niedersächsisches Landesamt für Verbraucherschutz und Lebensmittelsicherheit* (LAVES, permit number 42502-04-12/0769). Intestinal IRI was induced as per the method previously described [18]. Briefly, mice were anesthetized with Ketamine (100mg/ml, Albrecht, Germany) and Xylazine (2%, Bayer HealthCare, Germany) by *i*.*p*. injection (0.5 ml Ketamine + 0.1 ml Xylazine + 4.4 ml 0.9% NaCl; both 0.01 ml/g BW). Normothermia was maintained at 37°C using heat lamps (Philips, Germany), breathing air was supplemented with oxygen (Linde, Germany), and to prevent dehydration, 4ml/kg/h isotonic saline was injected subcutaneously. To induce ischemia, the mesenteric artery was clamped for 30min with spring-loaded bulldog microvascular clamps (Aesculap, Germany) after a midline laparotomy. Reperfusion was confirmed by observing pulsatile mesenteric flow before the abdominal cavity was closed. Between and after the abdominal manipulations, the abdominal cavity was closed with interrupted sutures (silk 8–0). After 4h of reperfusion, animals were sacrificed by cervical dislocation, and small intestinal tissue and serum samples were collected. Aged-matched animals underwent the same procedure without the mesenteric artery being clamped, and these served as controls in all experiments.

### mRNA extraction and assessment of cytokine expression by quantitative reverse transcription-polymerase chain reaction (qPCR)

mRNA extraction and the assessment of cytokine expression by quantitative PCR was performed as previously described [18,19]. In brief, total RNA was extracted from lysed gut tissue using the RNeasy Mini Kit (Qiagen, Venlo, The Netherlands) and complementary DNA was prepared using a high-capacity RNA-to-complementary DNA Kit (Applied Biosystems, Foster City, CA). All cDNA transcripts were assessed by QuantiTect Primer Assay (Qiagen) and Maxima SYBR Green/Rox qPCR MasterMix (Thermo Scientific, Waltham, MA) on an Applied Biosystems StepOnePlus Real-Time PCR System (Life Technologies, Carlsbad, CA). Relative gene expression values were normalized to hypoxanthine-guanine phosphoribosyltransferase (for cxcl1 and cxcl2) or glyceraldehyde-3-phosphate dehydrogenase (for il6 and tnf-a) as a housekeeping gene.

### Isolation of intestinal leukocytes

Intraepithelial leukocytes (IEL) and lamina propria leukocytes (LPL) were isolated as previously described: [18,19] The small bowel was removed from the abdominal cavity and flushed with cold PBS. The intestines were cut longitudinally to remove the feces. Fat, mesentery, and Peyer patches were removed. To isolate IEL, the intestine was cut into small pieces and incubated in PBS with 10% fetal calf serum (FCS) (PAA Laboratories, Cölbe, Germany) and 5 mM EDTA (Applichem, Darmstadt, Germany) for 20 minutes on an K-15 orbital shaker with incubation hood T-15 (Edmund Bühler GmbH, Hechingen, Germany) at 37°C and 200 revolutions per minute (rpm). After that, the samples were vortexed for 20s, and the supernatant was collected and stored on ice. The supernatants were poured through a 100 μM (BD, San Jose, USA) filter and a 40μM (BD) filter, centrifuged (5min, 300g, 4°C) and the collected cells were then re-suspended for FACS analysis. The pieces of the intestine remaining on the filter were used for isolation of lamina propria leukocytes by washing them in 10 mL RPMI 1640 media (GIBCO, Grand Island, USA) containing 10% FCS, followed by collagen digestion in 10 mL RPMI 1640 media supplemented with 10% FCS, 25 mM 4-(2-hydroxyethyl)-1-piperazineethanesulfonic acid (HEPES) (PAA Laboratories, Pasching, Austria) and 100 μg/mL Liberase (Roche, Basel, Switzerland) for 60min at 37°C and 200 rpm on an orbital shaker. The solution was centrifuged (5min, 300g, 4°C) and the pellet was resuspended in 40% Percoll (GE Healthcare, Buckinghamshire, United Kingdom). This solution was under-layered with an equal volume of 70% Percoll followed by Percoll gradient centrifugation (Acc: 9, Dec: 1, 460g, 40min, 25°C). The interphases were taken off and added to 10× their volume of 4°C PBS. After centrifugation (5 minutes, 300 g, 4°C), the cells were re-suspended in proper buffer for FACS staining.

### Flow cytometry

The absolute cell numbers were estimated, and the flow cytometric analyses were performed according to standard protocols as described previously [19,20]. In brief, fixable viability dye (eBioscience, Santa Clara, CA) was used to exclude dead cells and specific stainings were performed with the following antibody clones and conjugated fluorochromes (all eBiosciences) in the presence of anti-FcgRII/III mononuclear antibody (Clone: 2.4G2; Biolegend, San Diego, CA), for T cells: CD45-30-F11-eFluor450, CD3e-145-2C11-PerCP-Cy5.5, CD4-RM4-5-APC-eFluor780, CD8a-53-6.7-FITC, TCRβ-H57-597-APC, and TCRγδ-eBioGL3-PE, and for myeloid cells CD45-30-F11-eFluor450 CD11b-M1/70-APC-efluor780, CD11c-N418-PerCP-Cy5.5, Ly6-G-1A8-PE-Cy7, and Ly-6C-HK1,4-PE. Samples were acquired on a FACS Canto II flow cytometer (BD) and analyzed using FACS Diva software (BD) and KALUZA software (Beckman Coulter, Brea, CA). Myeloid subpopulations were assessed using the gating strategy as described by Rose et *al*. [21] and T cells were measured as previously described by our group [19].

### Assessment of liver enzymes and cytokines in serum

Alanine transaminase (ALT) and aspartate transaminase (AST) were measured using an AU 400 Olympus Analyzer (Olympus, Tokyo, Japan) as previously described [22]. TNF-α, IL-6, MIP-1α, and IFN-γ were measured using a cytometric bead-based (CBA) immunoassay (BD) according to the manufacturer’s instructions. Sample acquisition was performed using a FACS Canto II with FACS Diva software, and data analysis was conducted using FCAP array v3 software (Soft Flow Inc, Pecs, Hungary).

### Histology

Gut paraffin sections were stained with hematoxylin and eosin (H&E), and analyzed under light microscopy. Gut cryosections (2 μm) were prepared for immunohistochemistry and processed as previously described with the following primary antibodies rat anti-mouse GR-1, rat anti-mouse F4/80 and anti-CD3 (Clone: 145-2C11) (AbD Serotec, Oxford, UK) [23]. Cytospin preparations of 10^5^ cells were prepared (10 min,600 g) and stained according to May-Gruenwald-Giemsa and analyzed with an Olympus CKX41 light microscope (Shinjuku, Tokyo, Japan) [24].

### Statistics

Power-analysis was performed to assess the required sample size using SigmaStat (Systat Software, San Jose, CA, USA). Shigematsu et al. found a reduction of myeloperoxidase activity by ~66% following IRI. Therefore, we determined a relevant effect to be the reduction of neutrophils of at least 50% as the primary endpoint resulting in a calculated group size of n = 6 (Power 0,931, Difference in Means 0,500, Standard Deviation 0,200, Number of Groups 4, Alpha 0,0500) [14]. One-way analysis of variance with a Tukey’s multiple comparisons test was performed with GraphPad Prism v6.0 (GraphPad Software, San Diego, CA, USA). Data are displayed as mean and SD with p <.05 considered to be statistically significant.

## Results

### Acute intestinal IRI does not impact T cell fractions

**Fig 1:**
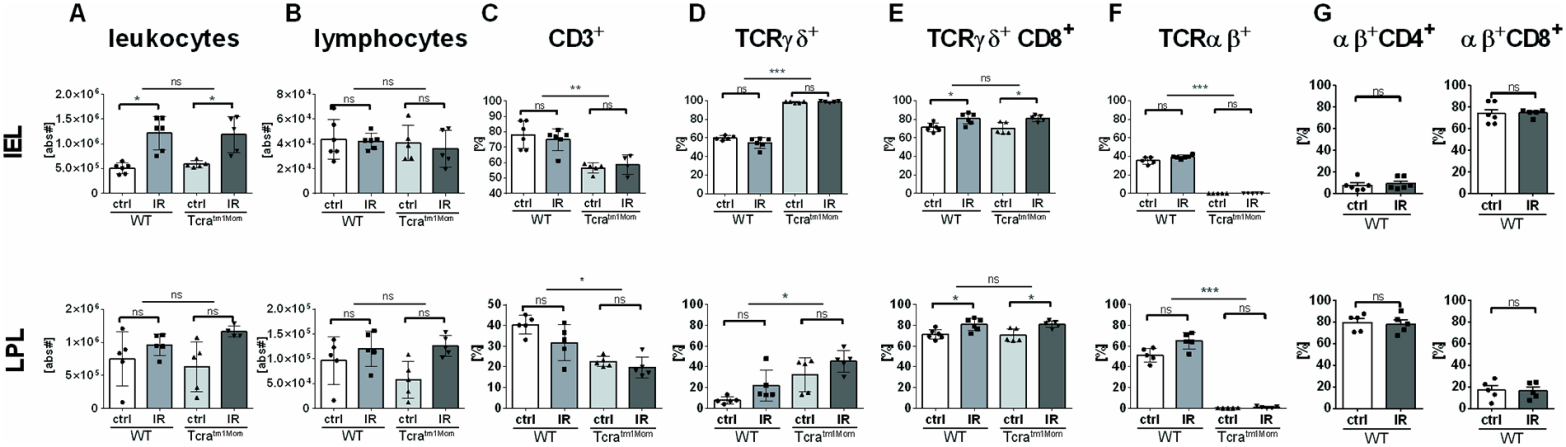
Acute intestinal IRI does not impact T cell fractions. A) Assessment of absolute leukocyte numbers (live CD45^+^ cells) in the epithelium of the small intestine (IEL, upper row) and lamina propria (LPL, lower row) of wild-type (WT) mice and mice deficient of αβ T cells (Tcra^tm1Mom^) after intestinal ischemia reperfusion (IR) or sham operation (ctrl). B) Absolute lymphocyte numbers were assessed examining the by forward and sideward scatter characteristics of live CD45^+^ cells in flow cytometric analyses. At least 1×10^5^ living leukocytes per sample were analyzed using flow cytometry. C) Percentage of total cells displayed in (B) positive for the pan T cell marker CD3. D) Percentages of CD3^+^ T cells positive for the gamma delta T cell receptor. E) Percentages of the gamma delta (γδ) T cells expressing CD8^+^. F) Percentages of alpha beta (αβ) T cells among CD3^+^ cells. G) Percentages of CD4 and CD8 subpopulations of αβ T cells in WT mice. Each data point represents the results of the analysis of an individual mouse. Bars represent group mean, and the error bar depicts ± SD. N = 5-6/group. ns = not significant. * = p<0.05; ** = p<0.01; *** = p<0.001. Data are representative of two independent experiments.

WT and αβ T cell deficient *Tcra*^*tm1Mom*^ mice were subjected to IRI or underwent a sham operation. All animals survived the procedure. The guts of all the mice that underwent clamping of the superior mesenteric artery exhibited macroscopic evidence of IRI with swelling, mucosal bleeding, and necrotic sections without any recognizable inter-individual differences. No perforation of the gut was observed. To gain further insights into the role of T cells play in acute intestinal IRI, we performed flow cytometric T cell-phenotyping of the lymphocytes isolated from the gut. The assessment of the total number of cells revealed the numbers of leukocytes in the intestinal epithelium had increased by approx. 2.5-fold following IRI in WT mice and *Tcra*^*tm1Mom*^ mice with no difference in regard to genotype (Fig 1A). The number of leukocytes isolated from the lamina propria was not statistically different (Fig 1A). For lymphocyte numbers, no significant differences could be found regarding induction of IRI or genotype by flow-cytometry (Fig 1B). This was visually confirmed by cytospin analyses (S1 Fig). T cell numbers among intraepithelial leukocytes (IEL) or lamina propria lymphocytes (LPL) did not change following IRI (Fig 1C). It is conceivable that the *Tcra*^*tm1Mom*^ mice displayed a significantly decreased numbers in their overall T cell fraction compared to WT animals due to the lack of αβ T cells (Fig 1C). In WT animals, about 2/3 of T cells expressed the gamma delta (γδ) T cell receptor, while 1/3 were αβ T cells (Fig 1D & 1F). Among the LPL, about 50–60% of T cells were αβ T cells in WT animals (Fig 1F). As expected, the T cell fraction in *Tcra*^*tm1Mom*^ mice consisted exclusively of γδ T cells (Fig 1D & 1F). Induction of IRI did not significantly alter these fractions (Fig 1D & 1F). Expression of CD8 on γδ T cells was not different between genotypes, but the induction of IRI significantly increased CD8^+^ γδ T cells (Fig 1E). Among IEL, CD8^+^ αβ T cells were predominant, while in the LPL fraction the majority of αβ T cells were CD4^+^ T helper cells (Fig 1G). Induction of IRI did not alter the fractions of CD4 or CD8 αβ T cells among IEL or LPL (Fig 1G).

### Neutrophils and inflammatory macrophages infiltrate gut after IR equally strong in WT and αβ T cell-deficient mice

**Fig 2:**
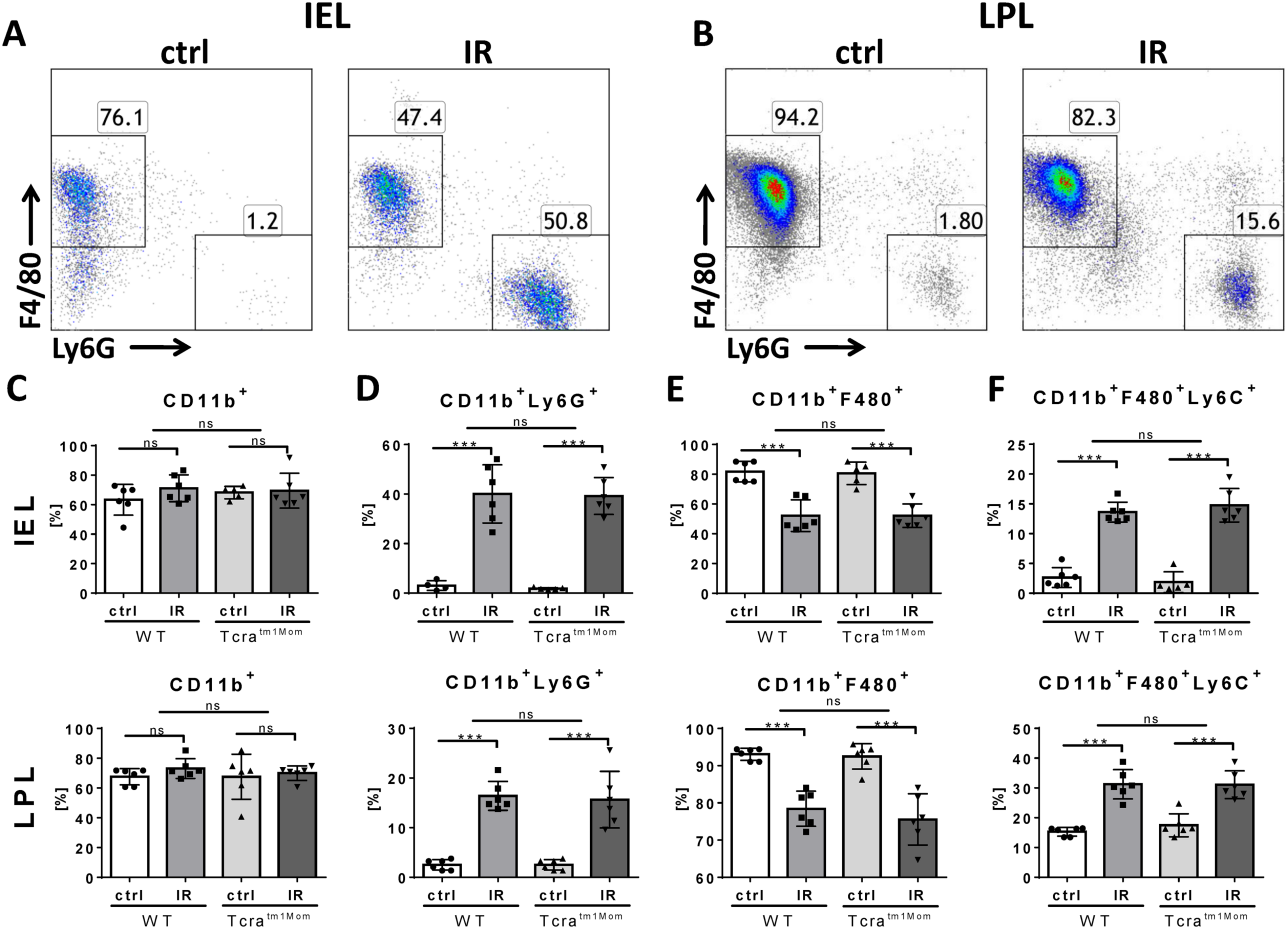
Neutrophils and inflammatory macrophages infiltration of the gut after IR was equally strong in WT and αβ T cell deficient mice. Intraepithelial leukocytes (IEL) and lamina propria leukocytes were isolated from small intestines and at least 1×10^5^ leukocytes per sample were analyzed using flow cytometry in wild-type (WT) mice and mice deficient of αβ T cells (Tcra^tm1Mom^) after intestinal ischemia reperfusion (IR) or sham operation (ctrl). Representative density plots of live CD45^+^CD11b^+^ leukocytes in IEL are displayed in (A), and LPL in (B) showing an increase of Ly6G^+^ neutrophils and a reciprocal relative decrease of F4/80^+^ macrophages. C) Percentage of CD11b^+^ myeloid cells in WT and Tcra^tm1/mom^ following IR or ctrl treatment. Data for IEL are displayed in the upper row, LPL in the lower row. D) Fraction of cells from (C) expressing the neutrophil specific marker Ly6G. E) Subgroup of cells from (C) expressing the macrophage marker F4/80. F) Percentages of macrophages expressing Ly6C indicating an inflammatory phenotype. Each data point represents the result of the analysis of an individual mouse. Bars represent group mean and error bar depicts ± SD. N = 6/group. ns = not significant. *** = p<0.001. Data are representative of two independent experiments.

In other organ models of IRI, neutrophils have been found to be a pivotal early cellular mediator of tissue damage, while monocytes/macrophages extend the immediate injury [25,26]. Using flow cytometry, we assessed the fraction of neutrophils and macrophages and identified a massive influx of Ly6G^+^ neutrophils into the intestinal epithelium (Fig 2A) and, to a lesser extent, into the lamina propria (Fig 2B). Following IRI, the relative proportion of CD11b^+^ myeloid cells among the (increased absolute leukocyte numbers) was unaltered (Fig 2C). However, the distribution of Ly6G^+^ neutrophils vs. F4/80^+^ macrophages among CD11b^+^ cells was reciprocal. Following IRI, a massive increase of neutrophils (Fig 2D) with a (relative) decrease of macrophages (Fig 2E) became apparent and was confirmed by immunohistochemistry (S2 Fig). However, no difference regarding genotype could be detected using flow cytometry (Fig 2C–2E) or cytospin analysis (S1 Fig). An assessment of the phenotype of macrophages revealed that the expression of Ly6C on macrophages increased following IRI, indicating an inflammatory phenotype (Fig 2F). However, there was no difference in regard to the genotype (Fig 2F). Assessment of CD11c^+^ populations in regard to Ly6G and F4/80 expression showed no differences among IEL or LPL following IRI in WT animals or αβ T cell deficient animals (S3 Fig).

### Up-regulation of pro-inflammatory cytokines is not altered by deficiency of αβ T cell

**Fig 3:**
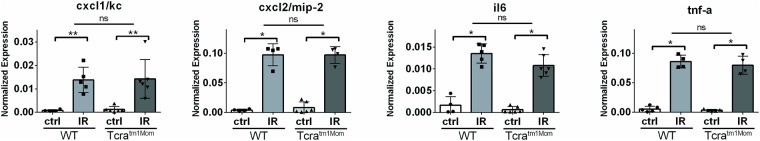
Neutrophils and inflammatory macrophages infiltrate gut after IR equally strong in WT and αβ T cell deficient mice. The relative expression of mRNA of CXCL1/KC, CXCL2/MIP-2, IL-6, and TNF-α in lysed tissue of small intestine of wild-type (WT) mice and mice deficient in αβ T cells after intestinal ischemia reperfusion (IR) or sham operation (ctrl) were assessed using quantitative reverse transcription PCR (RT-qPCR). The results are presented as the mean normalized expression value with GAPDH as the housekeeping gene. Data are represented as mean ± SD (n≥4). ns = not significant. *** = p<0.001. Data are representative of two independent experiments.

A key feature of IRI is an inflammatory response that is not only detrimental for the affected organ but also for the organism as a whole. With the exception of the αβ T cells, all investigated fractions of immune cells were not altered in *Tcra*^*tm1Mom*^ mice in comparison to WT mice. To identify possible effects a lack of αβ T cells had on inflammation, we assessed the expression of pro-inflammatory cytokines in the gut tissue by using qPCR. The chemokines CXCL1/KC and CXCL2/MIP-2 are major neutrophil attractants that are mainly secreted by monocytes and macrophages. IL-6 and TNF-α, as archetypical pro-inflammatory cytokines, are secreted by T cells and macrophages to regulate the inflammatory reaction during the acute phase response. As expected, the expression of messenger RNA for CXCL1/KC, CXCL2/MIP-2, IL-6, and TNF-α, were up-regulated following IRI of the gut (Fig 3). WT mice and αβ T cell-deficient mice demonstrated an equally strong up-regulation of these pro-inflammatory cytokines (Fig 3).

### Increase in transaminases and inflammatory cytokines in serum following intestinal IRI, with no effect of αβ T cell deficiency

**Fig 4:**
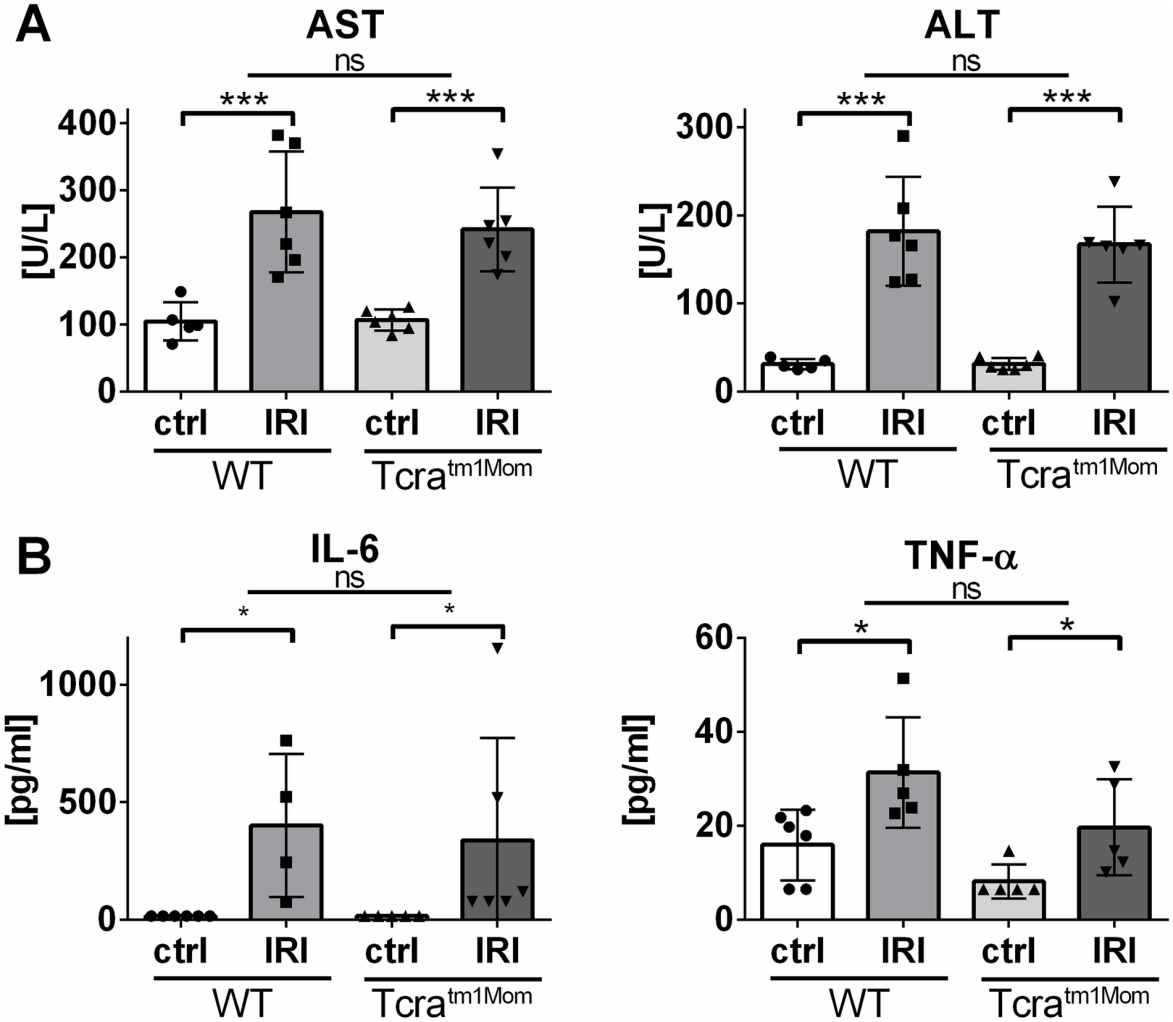
Increase of transaminases following intestinal IRI, with no effect of αβ T cell deficiency. Levels of serum transaminases AST and ALT (A) and IL-6 and TNF-α (B) of wild-type (WT) mice and mice deficient in αβ T cells (Tcra^tm1Mom^) after intestinal ischemia reperfusion (IR) or sham operation (ctrl). Each data point represents the results of the serum analysis of an individual mouse. The bars represent group mean and the error bar depicts SD. N = 5-6/group. ns = not significant. * = p<0.05, *** = p<0.001. Data are representative of two independent experiments.

The assessed immune cells and local inflammatory parameters in the gut indicated that the inflammation was not differentially regulated in *Tcra*^*tm1Mom*^ mice. To identify and evaluate systemic effects of intestinal IRI, we measured ALT and AST levels in the sera as markers of distant organ injury. Our results demonstrated that there was a significant up-regulation of both enzymes following intestinal IR (Fig 4). In WT animals, ALT increased significantly from 31.0 U/l (SD ± 5.8, lower 95% confidence interval (CI) of mean 23.8; upper CI 38.2) to 182.0 (± 61.7 U/L CI 117.3; 246.7) (Fig 4). AST increased from 104.4 U/l (± 28.3 CI 69.2; 139.6) to 267.7 (± 89.7 U/L CI 173.5; 361.9) (Fig 4). In αβ T cell-deficient mice, ALT increased from 31.3 ± 2.9 to 150.3 ± 42.0 U/L and AST from 106.8 U/L (± 15.7 CI 90.3; 123.3) to 241.8 U/L (± 62.4 CI 176.4;307.3), respectively (Fig 4A). As such, the results indicated that the deficiency of αβ T cells had no impact on the regulation of these injury markers. In addition, we assessed the serum levels of the pro-inflammatory cytokines TNF- α, IL-6, MIP-1α, and IFN-γ. The latter two were below the detection limit in all groups, while IRI resulted in significantly increased levels of IL-6 from 15 (± 5) pg/ml to 401 (± 152) and TNF-α from 15.9 (± 3.0) pg/ml to 31.3 (± 5.2) pg/ml. However, no significant differences between WT and αβ T cell-deficient mice could be found (Fig 4B).

## Discussion

The pathogenesis of IRI represents a complex interplay of numerous factors with inflammation as a common feature. The inflammatory response following IRI was initially thought to be mediated solely by tissue factors and the innate immune system. However, emerging data has demonstrated that T cells play a significant role as IRI mediators in various organs. T cells have been found to contribute to IRI in the brain, heart, liver, kidney, and limbs [1,9–12]. However, the role of T cells in intestinal IRI is less clear. In particular, a putative role of αβ T cells, the T cell fraction archetypical for the adaptive immune system, has not yet been studied in detail. Thus, this study aimed to clarify the extent to which αβ T cells contribute to intestinal IRI.

The research findings indicate that acute intestinal IRI has no influence on the absolute numbers of lymphocytes, the distribution of T cell fractions in the lamina propria, or the intestinal epithelium after four hours of reperfusion (Fig 1). This finding is largely in agreement with that of Qui et al., who focused on the IEL phenotype and demonstrated a slight increase in αβ T cells and a reciprocal decrease in γδ T cell fraction in response to IRI [13]. The effects observed in that study were rather mild with a relative change of 10% and the applied reperfusion time with 6 hours longer than in our model, thus may explaining the minor differences between their findings and that of the current study [13]. Nevertheless, the question of the T cell influx in intestinal IRI remain unresolved as influx kinetics in the literature are discrepant: One further study showed an increase of CD4^+^ (αβ) T cells in the gut 1h after ischemia (but not after 3 hours of reperfusion) compared to control mice, while another found no difference 1 hour after reperfusion but a peak of T cells after 6 hours of reperfusion [14,27]. We assume that the different mouse strains and different methodological approaches utilized in these studies may account for the inconsistencies. Further studies that adopt a more systematic approach are required to elucidate these observations.

In addition to the T cell distribution observed in the current study, we also confirmed previous findings of a strong neutrophil influx following intestinal IRI (Fig 2 and S1 and S2 Figs). Previous studies have found that neutrophils are early cellular mediators of tissue damage, while monocytes/macrophages extend the immediate injury [25,26,28]. These studies utilized myeloid peroxidase activity, histology, or flow cytometric assessments with the GR-1 antibody to demonstrate neutrophil involvement in the pathogenesis of intestinal IRI [25,26,28]. However, in addition to binding to the granulocyte specific epitope Ly-6G, GR-1 also binds to Ly6C, a marker of inflammatory macrophages [29]. Therefore, we used separate antibodies that were specific to each epitope to differentiate between the two populations via flow-cytometry [21]. This approach allowed us to identify an increase of absolute leukocyte numbers in intestinal IRI (Fig 1A), and to determine that this increase is caused by an influx of CD11b+ cells; i.e. myeloid cells, including neutrophils, and, to a lesser extent, inflammatory macrophages (Fig 2) [28].

As described in numerous other models, we identified an up-regulation of pro-inflammatory cytokines in the gut tissue following intestinal IRI (Fig 3) as well as an elevation of IL-6 and TNF-a in the serum (Fig 4) [10,11,28]. Additionally, we measured transaminases as marker of distant organ injury in intestinal IRI, which were significantly upregulated (Fig 4). As these serum markers are easily quantifiable it may represent a useful tool for future studies in the field. However, the exact molecular mechanisms of distant organ injury remain elusive. Studies with renal IRI suggested humoral as well as cellular factors to cause remote organ impairment [30]. The increase of IL-6 and to a lesser extent TNF-α in the serum following intestinal IRI (Fig 4) thus may cause the AST/ALT elevation by a systemic inflammatory response. It has also been demonstrated that renal IR induces oxidative stress in the liver resulting in hepatic dysfunction and also an increase in hepatic tumor necrosis factor levels and myeloperoxidase activities [31].

The key findings of our study were that the genetic deficiency of αβ T cells in intestinal IRI had no impact on the neutrophil influx, macrophage activation, up-regulation of pro-inflammatory cytokines, or the release of transaminases (Figs 1–4). These findings were, to some extent, surprising, as αβ T cells, particularly CD4^+^ T helper cells, are unequivocally recognized as key mediators in the pathogenesis of IRI in other organs [1,9–11]. Initially reported in liver and kidney models of IRI, the observed involvement of T cells in IRI has have been extended to heart, brain, and limb models and these are supported by multiple lines of evidence: 1) Immune suppressive agents ameliorate IRI of brain, kidney, and liver; 2) Blocking co-stimulatory molecules and, thus, inhibiting T cell activation or blocking the integrins necessary for homing into the damaged organ attenuate IRI in multiple organs; 3) Deficiency of T cells, either utilizing knock-out experiments or T cell depleting monoclonal antibodies, in addition with adoptive transfer experiments have firmly established T cells key actors in IRI of the brain, lung, myocardium, kidney, and liver [1,9–11]. However, in the applied model of intestinal IRI, αβ T cell deficiency did not result in any significant changes in any of our read-out parameters. This could be explained by the acute nature of the intestinal IRI model and the notion that T cells play a more significant role in IRI over a prolonged period. In contrast to kidney models, for example, intestinal IRI has only a relatively short reperfusion phase and no assessment of long-term effects possible due to the severity of the intestinal model [32]. Thus, due to the delayed mode of action of the adaptive immune response, possible long-term effects may be underestimated in the intestinal IRI model. An additional explanation is offered by the fact, that in comparison to other immune cells, αβ T cells are relatively rare in the gut. γδ T cells represent the majority of intraepithelial T cells and were not affected by our approach of selective genetic αβ T cells deficiency (Fig 1). Of course, this does not exclude the possibility that αβ T cells may play a role in delayed IRI response or chronic conditions following IRI in the gut. However, our data demonstrates that, in the first hours following intestinal IR, αβ T cells do not significantly contribute to the acute injury. Our findings are seemingly in opposition to existing studies that have suggested an involvement of T cells in intestinal IRI: Studies in SCID mice demonstrated protection from intestinal IRI [14,27,33]. Shigematsu and colleagues reported a significant reduction in the intestinal leakage of albumin compared to the WT and restoration of the WT levels of albumin extravasation was reported in SCID mice reconstituted with T cells from WT mice. Tissue myeloperoxidase activity was also significantly decreased in the SCID mice when compared to WT. The authors concluded that (all types of) T cells are important for recruitment of neutrophils and that T cells have a pathogenic effect during intestinal IRI [14]. Likewise, Edgerton et al. show amelioration in another strain of SCID mice, reconstitution of tissue damage following repletion of T cells, and mitigation of injury following anti-CD4-antibody treatment [27]. These major differences contrasting our results may be explained by the fact that both strains of SCID mice utilized do not only lack all T cell populations, including γδ T cells, but also B cells and immunoglobins (Ig). Concerning the latter, complement-dependent injury is central to IRI and mediated via activation of the complement cascades by antibodies [1,7,34]. Animals with a genetically selective deficiency of Ig exhibit a significantly reduced intestinal injury, while reconstitution with Ig completely restored the degree of intestinal injury [33,35]. Thus, the lack of (natural) immunoglobins (capable of recognizing self-antigens) may sufficiently explain the amelioration of intestinal IRI in SCID mice. In addition, SCID mice do not only lack αβ T cells but also γδ T cells: γδ T cells have pleiotropic effector functions including rapid innate-like immune responses particularly at mucosal borders [36,37]. Therefore, their lack in SCID mice, as opposed to isolated deficiency of αβ T cells in our study, offers another potential explanation for the conflicting findings. In one study, SCID mice were reconstituted with T cells negatively selected with a magnetic bead assay [27]. This may be explained by the overall low purity of this methodological approach and specifically by the fact that γδ T cells were also co-transferred. Further studies should clarify the role γδ T cells play in the gut, as they do make a significant contribution to IRI alternative models [38–40].

Another study that examined T cells as mediators in intestinal IRI found that the injury improved when polyclonal anti-thymocyte globulin (ATG) was administered [15]. However, ATG is a polyclonal mixture of antibodies that target various structures in the immune system on several different effector cells and also proteins that are essential for proper adhesion and homing to the site of inflammation [16]. Thus, the amelioration of intestinal IRI following ATG administration may also be explained by mechanisms other than targeting αβ T cells. Similarly, the findings of another study that demonstrated the amelioration of intestinal IRI following anti-CD4 injections may also have been affected in a similar manner [27]. Off-target inhibition of cells other than T cells, which also express the CD4 epitope (e.g. monocytes, macrophages, or dendritic cells) may account for the observed effects.

Collectively, we demonstrate that in the applied model of acute intestinal IR selective αβ T cell deficiency did not impact the severity of the injury in the gut. We conclude that, in the model of intestinal IRI, αβ T cells are functionally dispensable for initiating or perpetuating the initial inflammatory reaction. Further research is required to clarify the precise role T cells play in intestinal IRI and to better understand the interplay between these cells and the other immune cells in this model.

## Supporting information

S1 Fig Intraepithelial leukocytes were isolated from the small intestines of WT and ab T cell deficient mice following intestinal IRI or sham procedure. Slides show representative cytospins after Pappenheim staining. Proportions of lymphocytes, granulocytes, and macrophages were determined by light microscopy.
(TIF)

S2 Fig Representative H&E staining of WT mice subjected to IRI or sham procedure with epithelial swelling and damage, cellular infiltrate, and signs of immune cell activation as well as apoptosis (upper row). The immunohistological studies show constant numbers of CD3^+^ cells, a slightly decreased expression of F4/80 (tissue resident macrophages) and a strong increase of GR-1, which is expressed on neutrophils and activated macrophages. (GR-1, F4/80, or CD3 in red, DAPI positive nuclei in blue, autofluorescence of epithelial cells in green).
(TIF)

S3 Fig Representative flow cytometric assessment of CD11b^+^ and CD11c^+^ subpopulations of live CD45^+^ cells among intraepithelial leukocytes (IEL, upper two rows) and lamina propria (LPL, lower two rows) of a wild-type mouse after intestinal ischemia reperfusion (IR) or sham operation (ctrl). In the plots in the left column six (I-VI) different subpopulations were identified based on the expression of CD11b and CD11c. The six columns on the right display the expression of F4/80 and Ly6G in these subpopulations. No differences regarding genotype could be observed.
(TIF)
