# Supplementary figures and images for: Conventional alpha beta (αβ) T cells do not contribute to acute intestinal ischemia-reperfusion injury in mice
Source: PLoS One. 2017 Jul 13;12(7):e0181326. doi: 10.1371/journal.pone.0181326 (PMC5509314; doi:10.1371/journal.pone.0181326)

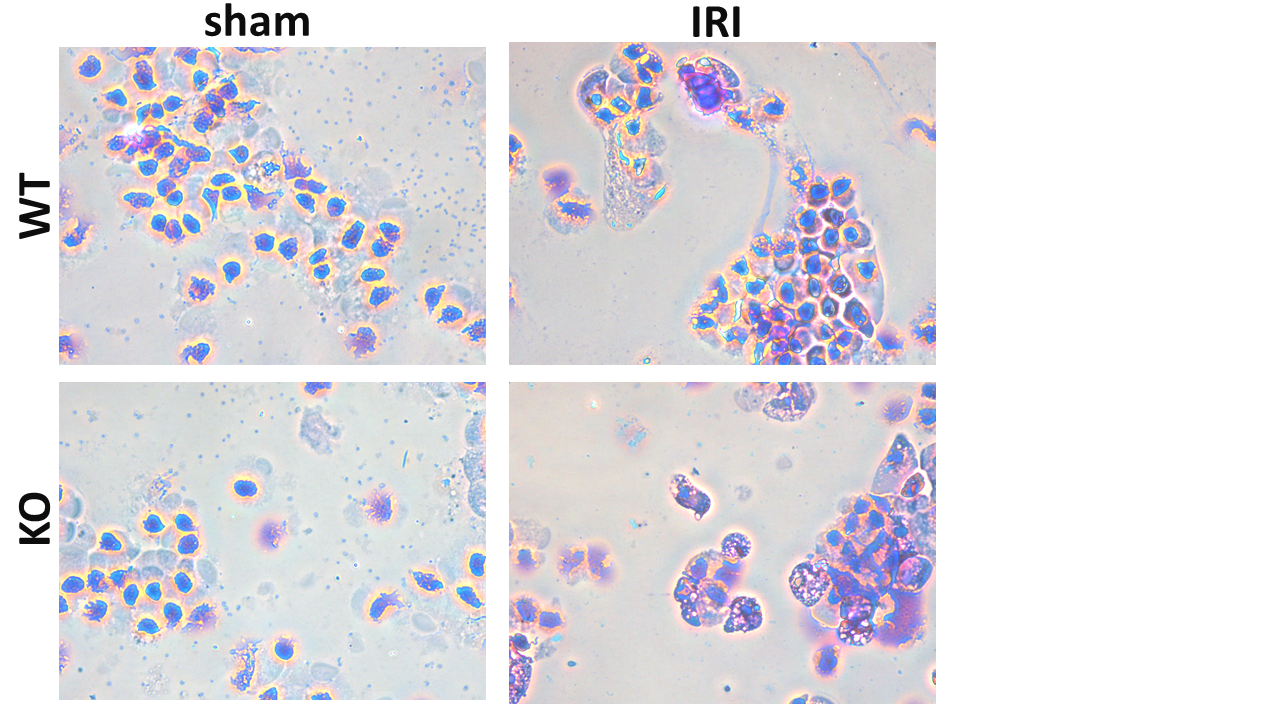

Supplement: S1 Fig — Slides show representative cytospins after Pappenheim staining. Proportions of lymphocytes, granulocytes, and macrophages were determined by light microscopy. (TIF) [file pone.0181326.s001.tif]

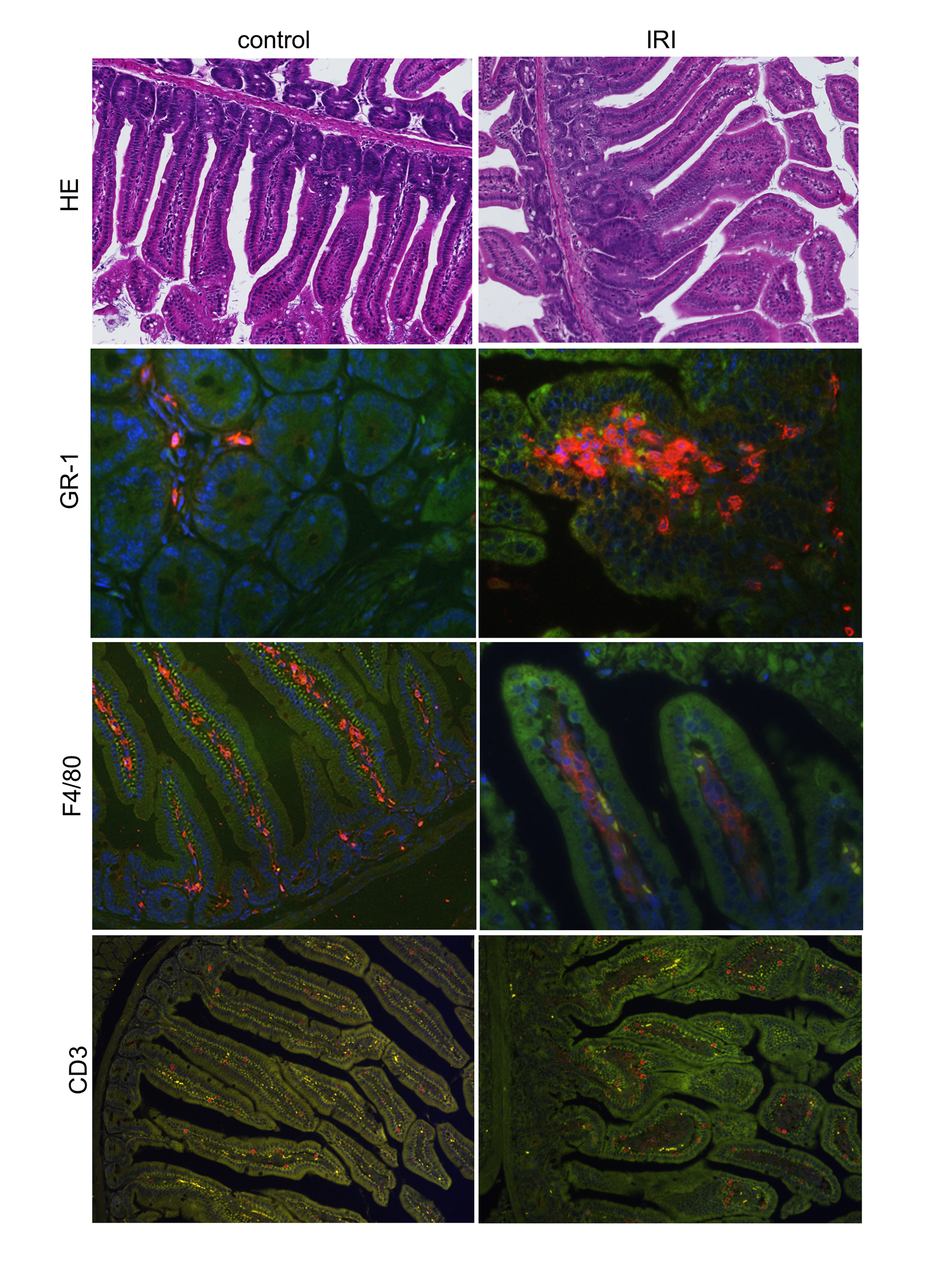

Supplement: S2 Fig — The immunohistological studies show constant numbers of CD3+ cells, a slightly decreased expression of F4/80 (tissue resident macrophages) and a strong increase of GR-1, which is expressed on neutrophils and activated macrophages. (GR-1, F4/80, or CD3 in red, DAPI positive nuclei in blue, autofluorescence of epithelial cells in green). (TIF) [file pone.0181326.s002.tif]

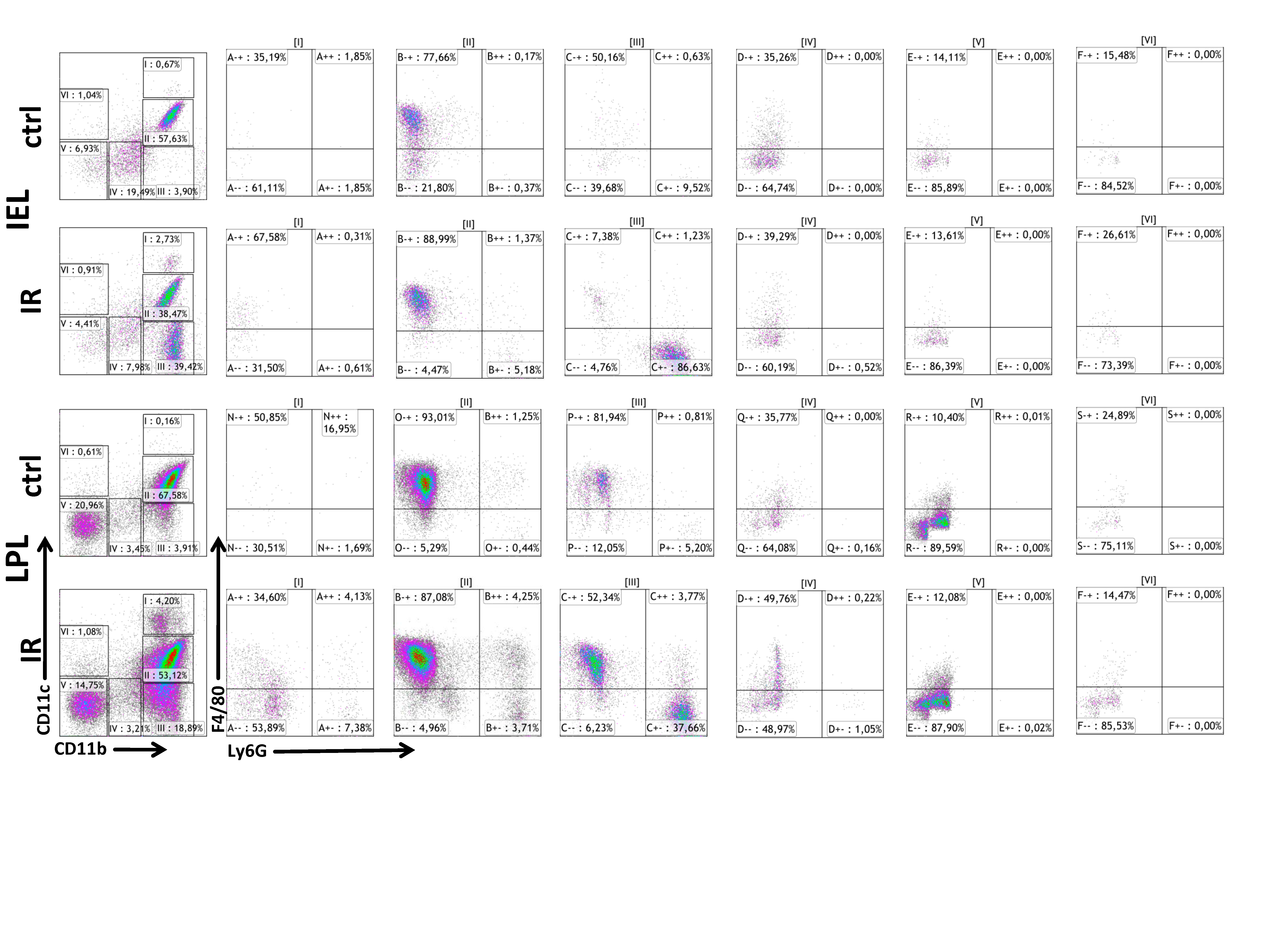

Supplement: S3 Fig — In the plots in the left column six (I-VI) different subpopulations were identified based on the expression of CD11b and CD11c. The six columns on the right display the expression of F4/80 and Ly6G in these subpopulations. No differences regarding genotype could be observed. (TIF) [file pone.0181326.s003.tif]
